# Binge Drinking, Alone or With Cannabis, During Adolescence Triggers Different Effects on Immediate Visual Memory in Men and Women

**DOI:** 10.3389/fpsyt.2021.797221

**Published:** 2021-12-16

**Authors:** Concepción Vinader-Caerols, Santiago Monleón

**Affiliations:** Department of Psychobiology, University of Valencia, Valencia, Spain

**Keywords:** binge drinking, cannabis, immediate visual memory, adolescents, sex

## Abstract

**Background:** This study examines the interaction between a history of binge drinking (BD), alone or with cannabis consumption, and the effects of acute alcohol exposure on immediate visual memory (IVM) (faces memory task, scenes memory task and IVM-IQ) in adolescents of both sexes.

**Method:** Two hundred and ninety adolescents, grouped into refrainers, binge drinkers and subjects with a history of simultaneous BD/Cannabis co-use, received a risk dose of alcohol or a control drink.

**Results:** Consumption Pattern (refrainers *vs*. binge drinkers *vs*. BD/Cannabis consumers) was not significant, while Treatment (acute alcohol *vs*. control drink) was significant in both sexes. Also, male binge drinkers' performance in the faces memory task was poorer than that of refrainers and BD/Cannabis consumers who consumed the control drink. BD/Cannabis consumers performed this task as capably as refrainers. In women, binge drinkers performed better than refrainers in scene memory and IVM-IQ tests when given alcohol, and binge drinkers performed worse than refrainers after consuming the control drink.

**Conclusions:** Acute alcohol consumption worsens IVM. Cannabis exerts a buffering effect in men. A cognitive tolerance effect is observed in women. Exposure during adolescence to alcohol, alone or with cannabis, can trigger different cognitive effects in men and women that could endure into adulthood.

## Introduction

We have previously observed differential effects of alcohol on memory performance in adolescent binge drinkers, with immediate visual memory (IVM) proving to be particularly sensitive to impairment by alcohol (1, 2). The prevalent pattern of alcohol use among adolescents and young adults in Western countries is binge drinking (BD), which has been defined by the National Institute on Alcohol Abuse and Alcoholism (NIAAA) as a pattern of drinking that raises a person's blood alcohol concentration (BAC) to 0.8 g/L or above (3). This pattern is characterized by intermittent consumption of large quantities of alcohol in short periods at intervals of between 1 week and 1 month and alternation between intoxication and withdrawal (4–6).

Researchers have investigated the effects of BD on different kinds of memory. Among them, word fragment completion, free recall, and IVM appear to be the most sensitive in adolescents and young adults, as they are affected by moderate doses of alcohol (BAC = 0.3 −0.38 g/L) [e.g., (2, 7)]. Higher doses of alcohol (BAC levels of BD, i.e., around 0.8 g/L) are necessary for this significant impairment to be observed with other types of memory, such as working memory [e.g., (1)] and short-term memory [e.g., (8)]. A plausible explanation for the lack of effects reported with BACs under 0.8 g/L [e.g., (8–10)] is that the brain of binge drinkers employs compensatory mechanisms in additional brain areas in order to perform tasks adequately, and that these resources are undermined by higher BACs [e.g., (11)].

On the other hand, according to a report from the Spanish Observatory of Drugs and Addictions (12), 13.4% of the Spanish adolescents who consumed alcohol during the last year also used cannabis, while 91.7 % of those who used cannabis also claimed to drink alcohol. This indicates that almost all cannabis users are co-users of alcohol.

The effects of alcohol and cannabis co-use on cognitive functions have been the subject of less research, and are more ambiguous than those of these substances when consumed alone. Some authors (13–15) consider that cannabis tempers the deteriorating effect of alcohol on cognitive functions, while others (16, 17) argue for a synergistic effect of cannabis on the deteriorating effect of alcohol on memory. In addition, there are no studies that have evaluated the phenomenon of cognitive tolerance in alcohol and cannabis co-users, a phenomenon observed in female alcohol users (2).

Experimental results of research performed with subjects of a single sex are sometimes extrapolated to both sexes. Sex should be considered as an important biological variable in basic and preclinical research, and results should not be automatically applied to both men and women (18). Sex differences in the effects of alcohol have been reported, supporting the view that the brains of male and female adolescents are differentially affected by alcohol use (19). There is evidence suggesting that female adolescents are more vulnerable to the neurotoxic effects of alcohol on cognition (19, 20), since the cognitive tolerance effect of alcohol on IVM develops in BD women but not in BD men (2). Several studies have demonstrated the differential associations of alcohol and cannabis co-use with the neurocognitive functioning of males and females, showing a different pattern of neurocognitive impairment in men and women [e.g., (21)]. In the light of these data, we deem it crucial to include both sexes in this study.

Taking into account the scarcity of studies evaluating the cognitive effects of alcohol and cannabis co-use in healthy adolescents, and considering the potential vulnerability of females to the neurotoxic effects of alcohol, the main objective of this research was to study experimentally the interaction between a history of BD, alone or simultaneously with cannabis use, and the effects of acute alcohol exposure on IVM (faces memory, scenes memory and IVM-IQ) in adolescents of both sexes. IVM was assessed by the Wechsler Memory Scale 3rd Edition (22), as it is a standard clinical instrument commonly used for evaluating this type of memory. Based on previous works, we already know that some long-term effects of repeated alcohol exposure in adolescents (such as alcohol tolerance or damaged cognitive abilities) are manifested more readily following ingestion of an acute dose of alcohol. Our hypotheses are: (1) A history of consumption and/or acute alcohol consumption would produce an impairment of IVM with sex-differential effects, being more evident in women than in men; (2) A synergistic effect could be observed vs. a buffering effect of cannabis on the detrimental effect of alcohol, which may also be sex-dependent.

## Methods

### Subjects

Two hundred and ninety 18–19 years old adolescent students (one hundred and eighteen males and one hundred and seventy-two females) from the University of Valencia, Spain, filled in a self-report questionnaire about physical and psychological health, frequency and level of consumption of alcohol, cannabis, alcohol with cannabis, or other drugs, and hours and quality of sleep. The participants were recruited for the study based on strict exclusion and inclusion criteria. The exclusion criteria were as follows: taking medication; a history of mental disorders (diagnosed by a health professional according to DSM criteria); an irregular sleep pattern (non-restorative sleep and/or irregular schedule); having consumed, even sporadically, any drug or having a history of substance use (including those studied herein); and having first-degree relatives with drug misuse problems. The following inclusion criteria were used: age 18–19 years old; a healthy body mass index (mean in men: 22.61 ± 0.24, mean in women: 21.55 ± 0.2); good health (without major medical problems); and being refrainers, occasional consumers of alcohol, binge drinkers or BD and cannabis co-users. The participants were classified as refrainers if they had never consumed alcoholic drinks or had drunk very sporadically. They were classified as alcohol consumers with a BD pattern in accordance with the NIAAA criteria for Spain [see (23)], i.e., if they had drunk six or more standard drink units (SDU = 10 g of alcohol) of distilled spirits (alcohol content ≥ 40 vol. %) in a short of period of time in the case of men, and five or more SDU in the case of women, at a minimum frequency of three occasions per month, throughout the previous 12 months (6). Finally, the participants were classified as co-users if they reported a pattern of BD along with cannabis use (i.e., they usually smoked a joint while they have an episode of BD). In fact, alcohol consumption is widespread among adolescents who use cannabis (in Spain, 91.7% who smoke cannabis also drink alcohol) (12).

The participants were told to follow their normal sleep pattern and meal routine, and to eat 1 h before the experimental session.

A telephone interview of approximately 15 min was conducted with each subject in order to confirm the information previously provided in the self-report and to fix a date and time for the test session. The participant's data were verified again on the test day. The data concerning the menstrual cycles of the female groups were registered in the self-report and during the telephone interview, and cycle phase was considered in the test in order to counterbalance this variable in each group.

### Test and Apparatus

The Alcohol Use Disorders Identification Test (AUDIT) (24) was employed to determine a problematic use of alcohol among the subjects. AUDIT is considered an appropriate screening instrument to classify BD and non-BD university students (25). The AUDIT consists of 10 questions that evaluate the quantity and frequency of alcohol intake, as well as alcohol-related behaviors and their consequences.

The Cannabis Abuse Screening Test (CAST) (26) is a six-item self-report that we employed to detect a problematic use of cannabis among the subjects during the previous 12 months.

A digital automatic blood pressure monitor (M10-IT, OMRON, Spain) was employed to register systolic and diastolic blood pressure and heart rate in all the subjects.

An alcoholmeter (Alcoquant® 6020, Envitec, Germany) was employed to evaluate BAC before and after (20 and 50 min) drink intake.

A drug test (DrugTest® 5000, Dräger, Spain) was employed to analyze the presence of drugs in a saliva sample collected from each participant before drink intake.

IVM was assessed using the Wechsler Memory Scale 3rd Edition (WMS–III; version adapted for the Spanish population) (22). The IVM subscales require the respondent to recognize faces (faces memory) and remember scenes (scenes memory). Specifically, in the faces memory task, the participants are shown 24 target faces, one at a time for 2 s. Then participants are shown 48 faces (24 targets and 24 distractors) and are asked to identify the target faces by responding either “yes” or “no” to each face. In the scenes memory task, the participants view four different scenes of four family members engaged in a common activity (such as buying clothing). After viewing all four scenes, the participants are shown a card divided into four quadrants, given the name of the scene, and asked to recall it, indicating where each family member was located in the original picture and what that family member was doing. Subjects' scores on the IVM scales (0–20 scalar scores were used both in face and scene memory tests) were transformed into centiles according to the subject's age to obtain the IQ of IVM.

### Procedure

The experimental procedure was approved by the Research Ethics Committee of the University of Valencia (Certification number: H1485172642673; approved on July 7th, 2017), and was in accordance with the Helsinki Agreement. All the subjects provided written informed consent to participate in the study. According to their Consumption Pattern (Refrainers, Binge Drinkers and BD/Cannabis consumers) and the Treatment received (Control drink and Alcohol), the participants were assigned to one of six experimental conditions for each sex: Refrainers-Control drink (R-Co); Refrainers-Alcohol (R-A); Binge Drinkers-Control drink (BD-Co); Binge Drinkers-Alcohol (BD-A); BD/Cannabis-Control drink (BD/C-Co) and BD/Cannabis-Alcohol (BD/C-A).

The participants were instructed to abstain from any intake of alcohol, caffeinated beverages, drugs or medication and strenuous exercise for 24 h before the experimental session, and to refrain from eating and smoking at least 1 h prior to the session. At the beginning of the experimental session BAC was measured in BD and co-user subjects using the alcoholmeter to ensure that they had not consumed alcohol. A saliva sample of the co-users was also analyzed by the drug test to make sure that they had not used other drugs.

In addition, a problematic use of alcohol or cannabis among the BD subjects and co-users was assessed using the AUDIT and CAST test, respectively. None of the subjects was found to be alcohol or cannabis-dependent (mean AUDIT: 8.35 ± 0.39 in men; 7.3 ± 0.28 in women; mean CAST: 2.83 ± 0.38 in men; 2.57 ± 0.28 in women). Next, each subject received a flavored refreshment (lime, orange or cola, without caffeine) contained in cans of 330 ml, alone or mixed (according to the experimental group) with distilled drinks of alcohol content = 40% vol. (vodka or gin) at a risk dose of 120 ml (equivalent to 38.4 g of alcohol). The subjects were instructed to consume their drink within a period of 20 min. The participants ate a light snack (the same for all the participants) and the beverages were always consumed in the presence of a research assistant. After finishing the drink, all the subjects rinsed their mouths with water, and BAC was repeatedly measured every 5 min throughout the waiting period, until it peaked (~20 min after consuming the drink). The subjects performed the IVM tests, faces memory test and scenes memory test, in a counterbalanced manner, while BAC was descending. The BAC of each experimental subject was measured again at end of the experiment.

BAC was 0.00 g/L for men and women before the alcoholic drink, and was 0.36 ± 0.008 g/L for men and 0.53 ± 0.01 g/L for women afterwards. It is important to point out that, although all the subjects consumed the same amount of alcohol, the statistical differences in BAC between men and women did not allow a direct comparison between the sexes. On the other hand, it was possible to study the effects of alcohol on each sex.

### Statistical Analyses

Data from men and women were analyzed separately, as BAC was found to be statistically different between the two sexes. The data were submitted to parametric analysis after checking that they met the criteria for normality and homogeneity of variances. Each analysis (faces memory, scenes memory and IVM-IQ) contained the between-subject factors Consumption Pattern (Refrainers, Binge Drinkers and BD/Cannabis consumers) and Treatment (Control drink and Alcohol). When their interaction was statistically significant, pairwise comparisons were carried out. All analyses were performed using the “SPSS” Statistics software package, version 26.0 for Windows (27).

## Results

### IVM in Men (BAC: 0.36 ± 0.008 g/L)

Table 1 shows a summary of descriptive statistics and significant differences observed among men with respect to Consumption Pattern and Treatment in the faces memory task, scenes memory task and IVM-IQ.

**Table 1:** Descriptive statistics for consumption pattern and treatment factors and significant differences observed in faces memory, scenes memory and IVM-IQ in men and women.

	**Consumption pattern**				**Treatment**		
**Men**	**Refrainers** **(*n* = 38)**	**Binge Drinkers** **(*n* = 50)**	**BD/Cannabis consumers (*n* = 30)**	**Statistical** **Power^a^ / Eta^**2**^**	**Control drink** **(*n* = 65)**	**Alcohol** **(*n* = 53)**	**Statistical Power^a^ / Eta^**2**^**
Faces memory	8.44 ± 0.37	7.44 ± 0.34	8.6 ± 0.57	0.352 / 0.03	8.75 ± 0.33	7.2 ± 0.32^***^	0.96 / 0.111
Scenes memory	9.39 ± 0.61	9.7 ± 0.51	8.76 ± 0.72	0.142 / 0.01	9.93 ± 0.42	8.66 ± 0.55	0.362 / 0.023
IVM-IQ	95.00 ± 2.35	92.780 ± 2.01	93.33 ± 3.11	0.096 / 0.005	97.36 ± 1.68	89.05 ± 2.12^**^	0.861 / 0.078
	**Consumption pattern**				**Treatment**		
**Women**	**Refrainers** **(*****n*** **=** **56)**	**Binge Drinkers** **(*****n*** **=** **80)**	**BD/Cannabis consumers (*****n*** **=** **36)**	**Statistical** **Power^a^** **/ Eta**^**2**^	**Control drink** **(*****n*** **=** **100)**	**Alcohol** **(*****n*** **=** **72)**	**Statistical Power^a^** **/ Eta**^**2**^
Faces memory	8.83 ± 0.39	8.61 ± 0.35	9.91 ± 0.47	0.182 / 0.009	9.91 ± 0.27	7.63 ± 0.35^***^	0.992 / 0.104
Scenes memory	9.33 ± 0.42	8.77 ± 0.38	9.02 ± 0.55	0.08 / 0.002	9.69 ± 0.3	8.06 ± 0.41^*^	0.761 / 0.042
IVM-IQ	95.94 ± 1.96	93.62 ± 1.58	98.16 ± 2.35	0.168 / 0.008	100.04 ± 1.28	88.79 ± 1.68^***^	0.995 / 0.111

*Results are expressed as mean ± SEM*.

a *Statistical Power was calculated using alpha = 0.05*.

* *p < 0.05*;

** *p < 0.005*;

*** *p < 0.001 vs. Control drink*.

#### Faces Memory

**Figure 1:**
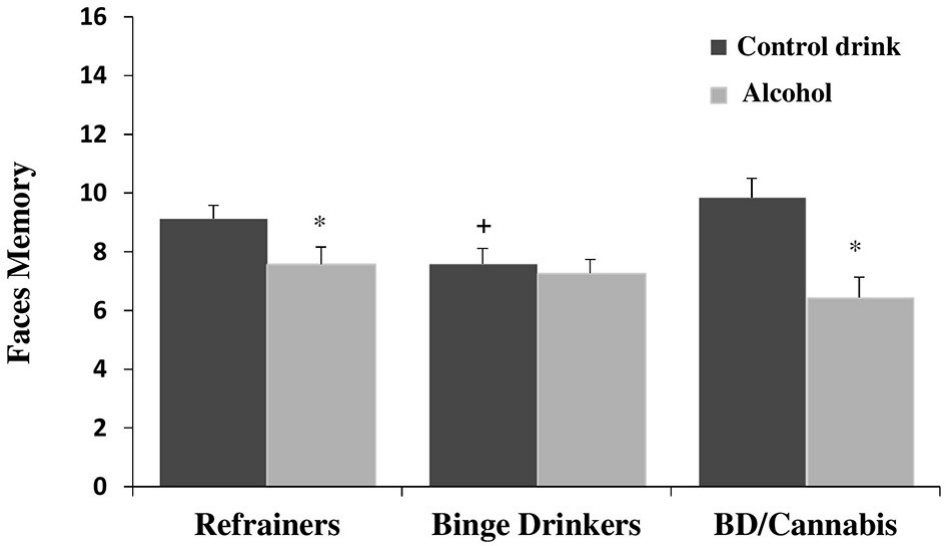
Performance in faces memory task (mean + SEM) in men. *p < 0.05 *vs*. Control drink. ^+^*p* < 0.05 *vs*. Refrainers-Control drink (R-Co) and BD/Cannabis-Control drink (BD/C-Co).

There was no significant main effect of Consumption Pattern on performance [*F*_(2, 112)_ = 1.705, ns]. The factor Treatment was statistically significant [*F*_(1, 112)_ = 14.007, *p* < 0.001], with subjects given alcohol showing lower scores than those given the control drink (see Table 1). The interaction Consumption Pattern X Treatment was also statistically significant [*F*_(2, 112)_ = 3.523, *p* < 0.05]. *Post hoc* comparisons showed that the R-A and BD/C-A groups performed worse than the R-Co and BD/C-Co groups, respectively (*ps* < 0.05); and that the BD-Co group performed worse than the R-Co and BD/C-Co groups (*ps* < 0.05), while no differences were detected between the last two groups (see Figure 1).

#### Scenes Memory

The factors Consumption Pattern [*F*_(2, 112)_ = 0.567, ns] and Treatment [*F*_(1, 112)_ = 2.622, ns] were not statistically significant, and neither was their interaction [*F*_(2, 112)_ = 0.812, ns].

#### IVM-IQ

There was no significant main effect of Consumption Pattern on performance [*F*_(2, 112)_ = 0.294, ns]. The factor Treatment was statistically significant [*F*_(1, 112)_ = 9.431, *p* < 0.005], with subjects given alcohol showing lower IVM-IQ than those given the control drink (see Table 1). The interaction Consumption Pattern X Treatment was not statistically significant [*F*_(2, 112)_ = 0.105, ns].

### IVM in Women (BAC: 0.53 ± 0.01 g/L)

Table 1 shows a summary of descriptive statistics and significant differences observed among women in terms of the factors Consumption Pattern and Treatment in the faces memory task, scenes memory task and IVM-IQ.

#### Faces Memory

There was no significant main effect of Consumption Pattern on performance [*F*_(2, 166)_ = 0.785, ns]. The factor Treatment was statistically significant [*F*_(1, 166)_ = 19.302, *p* < 0.001], with subjects given alcohol scoring lower than those given the control drink (see Table 1). The interaction Consumption Pattern X Treatment was not statistically significant [*F*_(2, 166)_ = 0.341, ns].

#### Scenes Memory

**Figure 2:**
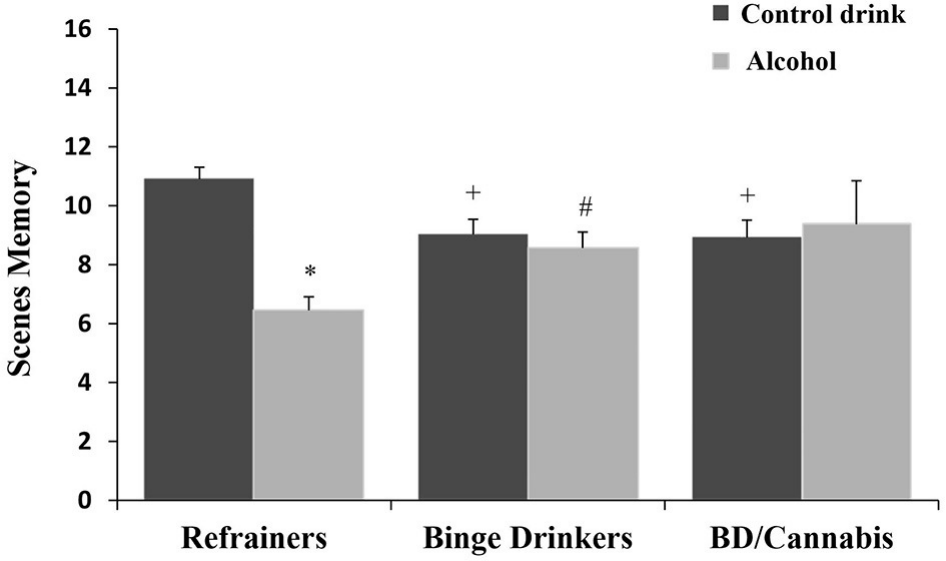
Performance in scenes memory task (mean + SEM) in women. *p < 0.05 *vs*. Control drink. ^+^*p* < 0.05 *vs*. Refrainers-Control drink (R-Co). ^#^*p* < 0.05 *vs*. Refrainers-Alcohol (R-A).

There was no significant main effect of Consumption Pattern on performance [*F*_(2, 166)_ = 0.182, ns]. The factor Treatment was statistically significant [*F*_(1, 166)_ = 7.203, *p* < 0.01], with subjects given alcohol obtaining lower scores than those given the control drink (see Table 1). The interaction Consumption Pattern X Treatment was also statistically significant [*F*_(2, 166)_ = 8.167, *p* < 0.001]; the R-A group performed worse than the R-Co group, and BD-Co and BD/C-Co participants performed worse than the R-Co subjects, while the BD-A group performed better than the R-A group (*ps* < 0.05) (see Figure 2).

#### IVM-IQ

**Figure 3:**
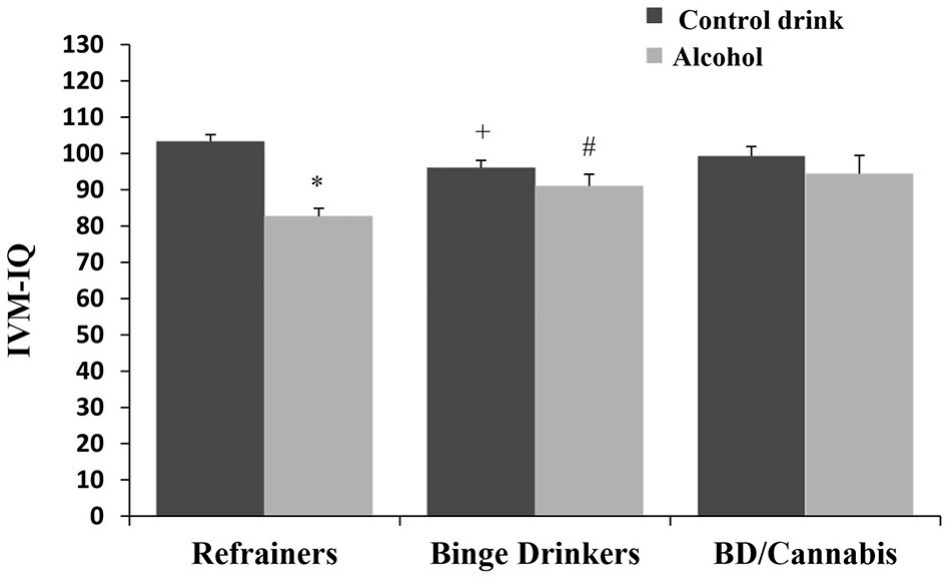
Immediate Visual Memory-IQ (mean + SEM) in women. *p < 0.05 *vs*. Control drink. ^+^*p* < 0.05 *vs*. Refrainers-Control drink (R-Co). ^#^*p* < 0.05 *vs*. Refrainers-Alcohol (R-A).

There was no significant main effect of Consumption Pattern on performance [*F*_(2, 166)_ = 0.707, ns]. The factor Treatment was statistically significant [*F*_(1, 166)_ = 20.733, *p* < 0.001], with subjects receiving alcohol displaying lower IVM-IQ than those given the control drink (see Table 1). The interaction Consumption Pattern X Treatment was also statistically significant [*F*_(2, 166)_ = 5.051, *p* < 0.01], with the R-A group performing worse than the R-Co group, the BD-Co group performing worse than R-Co subjects, and the BD-A group outperforming the R-A group (*ps* < 0.05) (see Figure 3).

## Discussion

A distinctive contribution of this study is to evaluate experimentally, together, the impact of an acute alcohol consumption episode and a BD history of consumption on IVM (faces memory, scenes memory and IVM-IQ) in adolescent men and women. We could not directly examine sex differences in IVM functioning among our adolescent population, but our study provides interesting data for each sex.

We have observed that a moderate acute dose of alcohol (BAC = 0.36 g/L in men and 0.53 g/L in women) is enough to impair faces memory and IVM-IQ in men, and faces and scenes memory and IVM-IQ in women, and corroborate that IVM is sensitive to the neurotoxic effects of acute alcohol consumption (2, 7). However, the maintenance of a BD pattern for 1 year did not affect IVM in any sex.

The literature suggests that this BD pattern and its maintenance over time have differential effects on memory (28). In the longitudinal study of Carbia et al. (28) executive difficulties disappeared after maintaining a BD pattern of alcohol consumption for 2 years (which was interpreted as an improvement), and the authors proposed than an alcohol-related delay in neuro-maturation, principally affecting prefrontal regions, resulted in BD subjects gaining executive efficiency later than age-matched non-BD individuals.

The pattern of alcohol and cannabis co-use did not affect the IVM in our study. Simultaneous alcohol and cannabis use in young drinkers (18–25 years old) has been associated with an increase of negative consequences (29). The existing literature on the effects of simultaneous alcohol and cannabis consumption on memory is scarce and contradictory. On the one hand, a synergistic deteriorating effect of alcohol and cannabis on cognitive processes has been reported (16, 17), with co-users being more likely to experience more severe cognitive consequences than users of alcohol alone (30). On the other hand, a buffering effect of cannabis against the deteriorating effects of alcohol on memory has been demonstrated by other researchers (13, 14). Cannabidiol (CBD) is a component of the cannabis plant with anti-inflammatory properties whose ability to improve cognitive impairment has been explored to an extent, but not conclusively. Nevertheless, authors such as Osborne et al. (31) have shown that CBD improves cognition in multiple preclinical models of cognitive impairment, including those of neuropsychiatric (schizophrenia), neurodegenerative (Alzheimer's disease), neuro-inflammatory (meningitis, sepsis and cerebral malaria) and neurological (hepatic encephalopathy and brain ischemia) disorders.

In line with the aforementioned evidence, we have observed, after a previous year of simultaneous consumption, a dampening effect of cannabis on the deteriorating effects of alcohol in the faces memory test when performed by men. After receiving the control drink, co-users performed this task as well as refrainers, while participants with a BD history performed worse than refrainers and co-users. It is plausible that the neuro-inflammatory effects of alcohol, responsible for cognitive decline, were counteracted by the anti-inflammatory efficacy of cannabis. Nevertheless, as other authors (14) have pointed out, long-term use of these substances can negatively increase vulnerability to the development of addictions. Furthermore, it must be taken into account the timeframe of consumption of these substances, as it is associated with the cognitive impairment (32).

In women, the interaction Consumption Pattern X Treatment was statistically significant with respect to scenes memory and IVM-IQ, and alcohol generated an effect of cognitive tolerance. This suggests that our binge drinkers developed tolerance in such a way that the deteriorating effect of alcohol on their scenes memory and IVM-IQ after drinking alcohol was weaker than that seen in refrainers. Thus, binge drinkers performed better than refrainers when given alcohol (displaying the abovementioned development of alcohol tolerance) and binge drinkers performed worse than refrainers after consuming a control drink (as their memory would have been damaged). The existence of this cognitive tolerance is endorsed by the fact that the scenes memory and IVM-IQ of female binge drinkers receiving the alcoholic drink did not differ from those of binge drinkers given the control drink. This phenomenon of cognitive tolerance was not so obvious in co-users, since there were differences in scenes memory between refrainers and co-users who received the control drink, but there were no differences in scenes memory or in IVM-IQ between refrainers and co-users who received alcohol. On the other hand, the phenomenon of women beginning to drink earlier and progressing more rapidly than men from the first exposure to the addiction phase, known as the “telescoping effect” (33–35), could explain why adolescent women develop cognitive tolerance earlier than men.

The alcohol acutely consumed by the participants in our study (38.4 g of alcohol) was close to the so called “risky alcohol consumption” for men and women (>5 SDU for men, and >4 SDU for women; 50 and 40 g of alcohol, respectively) (36). Some of the different effects observed in men and women in our study could be explained by the differences in BAC between the sexes in our population. It is possible that cognitive tolerance to alcohol would also be observed in male subjects with a higher BAC. This brings us to a limitation of our study, as the statistical differences in BAC between men and women did not allow a direct comparison between sexes. In this context, identifying gender-specific effects of alcohol and cannabis on male and female adolescents separately may help to explain differential proneness to substance use in adolescents (21). Acute alcohol consumption *vs*. a history of BD pattern leads to differential effects on cognition that depend on the type of memory in question. In conclusion, exposure during adolescence to alcohol, alone or with cannabis, can trigger different cognitive effects in men and women, which contribute to enduring cognitive deficits in adulthood. Moreover, our findings are consistent with the greater vulnerability of adolescent women to the neurotoxic effects of alcohol. Further research is needed–particularly, longitudinal studies including women and exploring the timeframe of consumption of these substances–in order to confirm the aforementioned findings and consolidate our conclusions. This will allow us to better understand the mechanisms underlying the effects on memory of alcohol, consumed alone or simultaneously with cannabis, and to develop optimal treatment methods for cannabis /alcohol dependence in men and women.

## Data Availability Statement

The raw data supporting the conclusions of this article will be made available by the authors, without undue reservation.

## Ethics Statement

The studies involving human participants were reviewed and approved by Research Ethics Committee of the University of Valencia (Certification number: H1485172642673; approved on July 7th, 2017). The patients/participants provided their written informed consent to participate in this study.

## Author Contributions

Both authors listed have made a substantial, direct, and intellectual contribution to the work and approved it for publication.

## Funding

This work was supported by the Universitat de València (Grant UV-INV_AE18-779336) and Generalitat Valenciana (Grant PROMETEO-II/2015/020), Spain.

## Conflict of Interest

The authors declare that the research was conducted in the absence of any commercial or financial relationships that could be construed as a potential conflict of interest.

## Publisher's Note

All claims expressed in this article are solely those of the authors and do not necessarily represent those of their affiliated organizations, or those of the publisher, the editors and the reviewers. Any product that may be evaluated in this article, or claim that may be made by its manufacturer, is not guaranteed or endorsed by the publisher.

## References

[1] Vinader-Caerols C Duque A Montañés A Monleón S. Blood alcohol concentration-related lower performance in immediate visual memory and working memory in adolescent binge drinkers. Front Psychol. (2017) 8:1720. 10.3389/fpsyg.2017.01720 29046656 PMC5632669

[2] Vinader-Caerols C Talk A Montañés A Duque A Monleón S. Differential effects of alcohol on memory performance in adolescent men and women with a binge drinking history. Alcohol Alcohol. (2017) 52:610–6. 10.1093/alcalc/agx040 28633431

[3] National Institute of Alcohol Abuse and Alcoholism (2004). NIAAA council approves definition of binge drinking. NIAAA Newsletter, 3:3.

[4] Maurage P Joassin F Speth A Modave J Philippot P Campanella S. Cerebral effects of binge drinking: respective influences of global alcohol intake and consumption pattern. Clin Neurophysiol. (2011) 123:892–901. 10.1016/j.clinph.2011.09.018 22055841

[5] Petit G Maurage P Kornreich C Verbanck P Campanella S. Binge drinking in adolescents: a review of neurophysiological and neuroimaging research. Alcohol. (2014) 49:198–206. 10.1093/alcalc/agt172 24302160

[6] Vinader-Caerols C Monleón S. Binge drinking and memory in adolescents and young adults. In: Palermo S Bartoli M editors. Inhibitory control training. A multidisciplinary approach. Valencia, Spain: IntechOpen (2019). 10.5772/intechopen.88485

[7] Söderlund H Parker ES Schwartz BL Tulving E. Memory encoding and retrieval on the ascending and descending limbs of the blood alcohol concentration curve. Psychopharmacology. (2005) 182:305–17. 10.1007/s00213-005-0096-2 16160875

[8] Brumback T Cao D King A. Effects of alcohol on psychomotor performance and perceived impairment in heavy binge social drinkers. Drug Alcohol Depend. (2007) 91:10–7. 10.1016/j.drugalcdep.2007.04.013 17560739 PMC2764986

[9] Rose AK Duka T. The influence of alcohol on basic motoric and cognitive disinhibition. Alcohol. (2007) 42:544–51. 10.1093/alcalc/agm073 17878213

[10] Spinola S Maisto SA White CN Huddleson T. Effects of acute alcohol intoxication on executive functions controlling self-regulated behavior. Alcohol. (2017) 61:1–8. 10.1016/j.alcohol.2017.02.177 28599712

[11] Day AM Celio MA Lisman SA Johansen GE Spear LP. Acute and chronic effects of alcohol on trail making test performance among underage drinkers in a field setting. J Stud Alcohol Drugs. (2013) 74:635–41. 10.15288/jsad.2013.74.635 23739029 PMC3711354

[12] Observatorio Español de las Drogas y las Adicciones (OEDA). Alcohol, tabaco y drogas ilegales en España (2019). Available online at: https://pnsd.sanidad.gob.es/profesionales/sistemasInformacion/informesEstadisticas/pdf/2019OEDA-INFORME.pdf

[13] Jacobus J McQueeny T Bava S Schweinsburg BC Frank LR Yang TT. White matter integrity in adolescents with histories of marijuana use and binge drinking. Neurotoxicol. Teratol. (2009) 31:349–355. 10.1016/j.ntt.2009.07.006 19631736 PMC2762024

[14] Jacobus J Squeglia LM Bava S Tapert SF. (2013). White matter characterization of adolescent binge drinking with and without co-occurring marijuana use: a 3-year investigation. Psychiatry Res. (2013) 214:374–81. 10.1016/j.pscychresns.2013.07.014 24139957 PMC3900025

[15] Mahmood OM Jacobus J Bava S Scarlett A Tapert SF. Learning and memory performances in adolescent users of alcohol and marijuana: interactive effects. J Stud Alcohol Drugs. (2010) 71:885–94. 10.15288/jsad.2010.71.885 20946746 PMC2965487

[16] Squeglia LM Gray KM. Alcohol and drug use and the developing brain. Curr Psychiatry Rep. (2016) 18:46. 10.1007/s11920-016-0689-y 26984684 PMC4883014

[17] Cummings C Beard C Habarth JM Weaver C Haas A. Is the sum greater than its parts? Variations in substance-related consequences by conjoint alcohol-marijuana use patterns. J Psychoactive Drugs. (2019) 51:351–9. 10.1080/02791072.2019.1599473 31002291

[18] Lee SK. (2018). Sex as an important biological variable in biomedical research. BMB Reports. (2018) 51:167–73. 10.5483/BMBRep.2018.51.4.034 29429452 PMC5933211

[19] Alfonso-Loeches S Pascual M Guerri C. Gender differences in alcohol-induced neurotoxicity and brain damage. Toxicology. (2013) 311:27–34. 10.1016/j.tox.2013.03.001 23500890

[20] Squeglia LM Schweinsburg AD Pulido C Tapert SF. Adolescent binge drinking linked to abnormal spatial working memory brain activation: differential gender effects. Alcohol Clin Exp Res. (2011) 35:1831–41. 10.1111/j.1530-0277.2011.01527.x 21762178 PMC3183294

[21] Noorbakhsh S Afzali MH Boers E Conrod PJ. Cognitive function impairments linked to alcohol and cannabis use during adolescence: a study of gender differences. Front Hum Neurosci. (2020) 14:95. 10.3389/fnhum.2020.00095 32317950 PMC7154290

[22] Wechsler D. Wechsler Adult Intelligence Scale-III. San Antonio, EEUU: The Psychological Corporation (2004).

[23] López-Caneda E Rodríguez Holguín S Corral M Doallo S Cadaveira F. Evolution of the binge drinking pattern in college students: neurophysiological correlates. Alcohol. (2014) 48:407–18. 10.1016/j.alcohol.2014.01.009 24835220

[24] Saunders JB Aasland OG Babor TF de la Fuente JR Grant M. Development of the Alcohol Use Disorders Identification Test (AUDIT): WHO collaborative project on early detection of persons with harmful alcohol consumption: II. Addiction. (1993) 88:791–804. 10.1111/j.1360-0443.1993.tb02093.x 8329970

[25] Cortés MT Giménez JA Motos-Sellés P Sancerni MD Cadaveira F. The utility of the Alcohol Use Disorders Identification Test (AUDIT) for the analysis of binge drinking in university students. Psicothema. (2017) 29:229–35. 10.7334/psicothema2016.271 28438247

[26] Legleye S Karila L Beck F Reynaud M. Validation of the CAST, a general population cannabis abuse screening test. J Subst Use. (2007) 12:233–42. 10.1080/14659890701476532

[27] IBM SPSS Statistics for Windows [computer program]. (2019) Version 26.0. Armonk, NY: IBM Corp.

[28] Carbia C Cadaveira F Caamaño-Isorna F Rodríguez-Holguín S Corral M. Binge drinking during adolescence and young adulthood is associated with deficits in verbal episodic memory. PLoS ONE. (2017) 12:e0171393. 10.1371/journal.pone.0171393 28152062 PMC5289570

[29] Linden-Carmichael AN Van Doren N Masters LD Lanza ST. Simultaneous alcohol and marijuana use in daily life: implications for level of use, subjective intoxication, and positive and negative consequences. Psychol Addict Behav. (2020) 34:447–53. 10.1037/adb0000556 31971426 PMC7148190

[30] Jackson KM Sokolovsky AW Gunn RL White HR. Consequences of alcohol and marijuana use among college students: Prevalence rates and attributions to substance-specific versus simultaneous use. Psychol Addict Behav. (2020) 34:370–81. 10.1037/adb0000545 31944787 PMC7064425

[31] Osborne AL Solowij N Weston-Green KA. (2017). Systematic review of the effect of cannabidiol on cognitive function: Relevance to schizophrenia. Neurosci Biobehav Rev. (2016) 72:310–24. 10.1016/j.neubiorev.2016.11.012 27884751

[32] Toledo-Fernández A Marín-Navarrete R Villalobos-Gallegos L Salvador-Cruz J Benjet C Roncero C. Exploring the prevalence of substance-induced neurocognitive disorder among polysubstance users, adding subjective and objective evidence of cognitive impairment. Psychiatry Res. (2020) 288:112944. 10.1016/j.psychres.2020.112944 32339804

[33] Piazza NJ Vrbka JL Yeager RD. Telescoping of alcoholism in women alcoholics. Int J Addict. (1989) 24:19–28. 10.3109/10826088909047272 2759762

[34] Johnson PB Richter L Kleber HD McLellan AT Carise D. Telescoping of drinking-related behaviors: gender, racial/ethnic, and age comparisons. Subst. Use Misuse. (2005) 40:1139–51. 10.1081/JA-200042281 16040374

[35] Sugarman DE Demartini KS Carey KB. Are women at greater risk? An examination of alcohol-related consequences and gender. Am J Addict. (2009) 18:194–7. 10.1080/10550490902786991 19340637 PMC2951597

[36] Mota N Álvarez-Gil R Corral M Rodríguez Holguín S Parada M Crego A. (2010). Risky alcohol use and heavy episodic drinking among Spanish University students: a two-year follow-up. Gac Sanit. (2010) 24:372–7. 10.1016/j.gaceta.2010.02.013 20656378

